# MIRA-CAP: Memory-Integrated Retrieval-Augmented Captioning for State-of-the-Art Image and Video Captioning

**DOI:** 10.3390/s24248013

**Published:** 2024-12-15

**Authors:** Sabina Umirzakova, Shakhnoza Muksimova, Sevara Mardieva, Murodjon Sultanov Baxtiyarovich, Young-Im Cho

**Affiliations:** 1 Department of Computer Engineering, Gachon University, Sujeong-gu, Seongnam-si 13120, Republic of Korea; sabinatuit@gachon.ac.kr (S.U.); sevara1998@gachon.ac.kr (S.M.); 2 Department of Information Systems and Technologies, Tashkent State University of Economics, Tashkent 100066, Uzbekistan; murad.sultanov@tsue.uz

**Keywords:** image and video captioning, vision–language models, streaming decoder, human-aligned evaluation

## Abstract

Generating accurate and contextually rich captions for images and videos is essential for various applications, from assistive technology to content recommendation. However, challenges such as maintaining temporal coherence in videos, reducing noise in large-scale datasets, and enabling real-time captioning remain significant. We introduce MIRA-CAP (Memory-Integrated Retrieval-Augmented Captioning), a novel framework designed to address these issues through three core innovations: a cross-modal memory bank, adaptive dataset pruning, and a streaming decoder. The cross-modal memory bank retrieves relevant context from prior frames, enhancing temporal consistency and narrative flow. The adaptive pruning mechanism filters noisy data, which improves alignment and generalization. The streaming decoder allows for real-time captioning by generating captions incrementally, without requiring access to the full video sequence. Evaluated across standard datasets like MS COCO, YouCook2, ActivityNet, and Flickr30k, MIRA-CAP achieves state-of-the-art results, with high scores on CIDEr, SPICE, and Polos metrics, underscoring its alignment with human judgment and its effectiveness in handling complex visual and temporal structures. This work demonstrates that MIRA-CAP offers a robust, scalable solution for both static and dynamic captioning tasks, advancing the capabilities of vision–language models in real-world applications.

## 1. Introduction

Generating accurate, contextually relevant captions for images and videos has become a crucial task in various real-world applications, from assistive technologies and video summarization to content recommendation and social media tagging [1]. Effective image and video captioning requires an understanding that goes beyond object recognition, delving into the interactions between scene elements, complex relationships, and the sequencing of events to produce coherent descriptions [2]. Despite recent advancements in vision–language models and attention mechanisms, which have elevated the performance of caption generation systems, challenges remain in achieving high-quality captions that align closely with human expectations, particularly in real-time video settings where scenes are dynamic and temporally complex [3].

Traditional approaches to captioning, such as encoder–decoder frameworks with attention mechanisms, have shown potential by enabling models to focus on specific image regions or video frames [4]. However, these methods face limitations, especially when used in dense video captioning tasks. Many models generate captions without a robust mechanism for maintaining long-term contextual coherence, resulting in fragmented or temporally inconsistent descriptions [5]. Additionally, models trained on large, web-sourced datasets are often hindered by noisy or irrelevant image–text pairs, leading to inaccurate captions [6]. The dynamic nature of streaming videos also poses a challenge, as current models are generally optimized to process entire videos at once, making them unsuitable for real-time applications where continuous, low-latency captioning is essential [7]. In response to these challenges, we propose Memory-Integrated Retrieval-Augmented Captioning (MIRA-CAP), a novel captioning framework that introduces a memory retrieval system to support contextual coherence, adaptive dataset pruning to mitigate noise in training data, and a streaming decoder for real-time video captioning. MIRA-CAP is designed to address the gaps in both image and video captioning by integrating these components, allowing it to generate semantically rich, temporally aligned captions that meet the needs of both static and dynamic scenarios. The cross-modal memory bank in MIRA-CAP serves as an innovative retrieval mechanism, enabling the model to access relevant embeddings from previous frames or captions. This memory retrieval approach allows the model to maintain a long-term memory of past context, crucial for producing descriptions that are temporally consistent and aligned across frames. By retrieving only the most contextually relevant memory embeddings, MIRA-CAP captures and reinforces the flow of events in dense video captioning, ensuring that each generated caption builds coherently on preceding frames. To address the challenge of training data noise, MIRA-CAP employs an adaptive dataset pruning technique that leverages synthetic captioning. This mechanism generates reference captions for each image and assesses their alignment with existing captions in the dataset, filtering out misaligned pairs while preserving the diversity necessary for effective generalization. By focusing on data alignment without sacrificing diversity, the MIRA-CAP pruning approach enhances the quality of the training data, allowing the model to learn accurate descriptions from a more representative dataset. For real-time video captioning, MIRA-CAP incorporates a streaming decoder that processes each frame sequentially, producing captions at designated decoding points. This streaming capability enables MIRA-CAP to handle long video sequences without requiring access to the entire video, making it suitable for applications that need continuous, low-latency captioning. By clustering past frames into memory clusters, the streaming decoder maintains a compact, informative memory representation that ensures the model can generate timely and contextually relevant captions even in live-streaming environments. In addition to the memory retrieval, pruning, and streaming components, MIRA-CAP utilizes a dual-attention mechanism that allows the model to focus selectively on both visual and textual information. This attention mechanism helps the model to balance immediate visual details with contextual memory, enabling it to produce captions that are detailed, coherent, and semantically aligned with the content.

MIRA-CAP introduces several innovations that set it apart from current state-of-the-art techniques in image and video captioning. Traditional memory-augmented models often rely on large, static memory banks that lack selective retrieval mechanisms, leading to redundancy and irrelevant content in captions. MIRA-CAP addresses this with its cross-modal memory bank, which retrieves only the most contextually relevant embeddings based on semantic similarity. This ensures that the generated captions are temporally coherent and grounded in meaningful historical context. Regarding dataset preprocessing, existing models typically use heuristic-based or static filtering methods that often discard valuable, diverse data. MIRA-CAP adaptive dataset pruning mechanism overcomes this limitation by generating synthetic captions to evaluate the semantic alignment of image–text pairs. This approach retains the diversity essential for generalization while eliminating noisy or irrelevant pairs, ultimately leading to improved caption quality and model robustness. Another significant distinction lies in MIRA-CAP’s streaming decoder, which is specifically designed for real-time video captioning. Unlike conventional models that require access to an entire video sequence before generating captions, MIRA-CAP processes video frames sequentially, producing captions incrementally. Employing clustering-based memory compression ensures that memory usage remains efficient even for extended video sequences. This capability makes MIRA-CAP particularly suitable for applications requiring continuous and low-latency captioning, such as live video feeds. Additionally, MIRA-CAP employs a dual-attention mechanism, effectively integrating visual and textual features. While traditional models often focus on a single modality, MIRA-CAP’s approach ensures that the generated captions are semantically rich and contextually aligned by attending to both the immediate visual scene and relevant historical context. This reduces the risk of hallucination and enriches the descriptive depth of captions. The evaluation framework further highlights MIRA-CAP advancements. While traditional metrics like BLEU and CIDEr are widely used, they often fail to capture nuanced semantic and contextual relationships. MIRA-CAP incorporates the Polos metric, which is designed to align closely with human judgment. By leveraging Polos in its feedback loop, MIRA-CAP dynamically adjusts its parameters during training to minimize errors such as hallucination and temporal misalignment, ensuring captions remain accurate and coherent. MIRA-CAP innovations in memory retrieval, dataset pruning, real-time processing, attention mechanisms, and evaluation metrics collectively address the limitations of current state-of-the-art techniques. These contributions make it a robust and scalable solution for generating high-quality captions across both static and dynamic scenarios.

The MIRA-CAP framework thus makes several significant contributions to the field of vision–language models. By introducing a memory retrieval system, the model enhances temporal consistency and contextual coherence in dense video captioning. The adaptive dataset pruning approach effectively filters noisy data, resulting in improved model generalization. The streaming decoder equips MIRA-CAP for real-time applications by allowing it to process and caption frames incrementally, while the dual-attention mechanism enriches the model’s ability to capture both visual and contextual elements. Through extensive experiments on standard datasets, such as MS COCO, YouCook2, and ActivityNet, we demonstrate MIRA-CAP’s ability to achieve state-of-the-art performance, evidenced by its high scores on metrics such as CIDEr, SPICE, and the Polos metric, which aligns closely with human judgment. The following sections will detail the technical foundations and experimental results of MIRA-CAP. In Section 2, we discuss related work on image and video captioning, memory-augmented models, and data pruning techniques. Section 3 describes the methodology behind MIRA-CAP, covering the cross-modal memory bank, adaptive dataset pruning, and streaming decoder in depth. Section 4 outlines the experimental setup, including datasets, implementation details, and baseline comparisons. Section 5 presents the results and analysis, with both quantitative and qualitative performance evaluation and ablation studies. Finally, Section 6 concludes with a summary of findings and future research directions.

## 2. Related Work

This section situates MIRA-CAP within the context of existing research on image and video captioning, memory-augmented models, dataset pruning, and evaluation metrics. By highlighting the limitations of prior approaches and the innovations that MIRA-CAP brings to address these gaps, this section provides a clear foundation for understanding the model contributions.

### 2.1. Image and Video Captioning

Image and video captioning have become fundamental tasks in the field of vision–language models, requiring models to interpret visual data and generate meaningful text descriptions [8]. Early works in image captioning focused on convolutional neural networks (CNNs) paired with recurrent neural networks (RNNs) in encoder–decoder architectures. The authors in [9] introduced visual attention mechanisms, allowing models to focus on specific image regions when generating each word in a caption, significantly improving the semantic alignment between images and text descriptions. For video captioning, additional challenges arise due to the temporal complexity of video content. Early approaches extended image captioning frameworks by introducing RNNs to sequentially process frames, yet they struggled with maintaining coherence across longer sequences [10]. To address these issues, researchers developed models with temporal attention and hierarchical structures, allowing for multi-scale processing of video content [11]. Recent approaches, such as transformer-based models, have further enhanced video captioning by using multi-head self-attention to capture long-range dependencies and relationships across frames, as demonstrated in Vid2Seq [12] and MeaCap [13] models. However, these models often process the entire video sequence as a single input, making them unsuitable for real-time or streaming applications where captions are needed incrementally. MIRA-CAP builds on this foundation by introducing a streaming decoder specifically designed for real-time video captioning. Unlike existing models that require access to the full video sequence, MIRA-CAP incrementally processes frames and produces captions at designated decoding points. This streaming approach aligns well with applications that require continuous, low-latency captioning, such as video conferencing or surveillance. Additionally, the MIRA-CAP dual-attention mechanism enables it to balance the immediate visual content with prior contextual information, thereby enhancing the coherence and relevance of generated captions over extended video sequences.

### 2.2. Memory-Augmented Models

Memory-augmented neural networks have emerged as a solution for tasks requiring long-term context and sequential coherence, including language modeling, machine translation, and video understanding [14]. Memory networks introduced by [15] allowed for external memory storage and retrieval, facilitating models to remember and reference past information relevant to the current input. Such memory systems have been particularly beneficial in domains where events span long sequences, requiring contextual connections across time. In the context of vision–language models, memory-augmented architectures have been applied to video captioning to address challenges of temporal alignment and coherence. Ref. [16] proposed a memory-augmented video captioning framework that integrates past frame representations to maintain continuity over time. However, these models generally rely on large memory banks, which can become computationally intensive and challenging to manage effectively over long video sequences [17]. Most memory-based captioning models also lack a sophisticated mechanism to selectively retrieve only the most contextually relevant information, resulting in redundancy or irrelevant content in generated captions. MIRA-CAP introduces a cross-modal memory bank with a retrieval mechanism tailored for image and video captioning tasks. By employing semantic similarity to retrieve only the most relevant memory entries, MIRA-CAP ensures that prior frames are referenced meaningfully and in a way that supports coherent, temporally aligned captioning. Furthermore, the model uses a clustering-based memory compression technique to maintain a fixed memory size, making it feasible to apply MIRA-CAP to arbitrarily long videos without the typical memory overhead. This approach helps MIRA-CAP deliver captions that are contextually grounded and sequentially coherent, addressing limitations in prior memory-augmented models.

### 2.3. Dataset Pruning Techniques

Large-scale datasets are essential for training vision–language models, yet they often contain noisy, misaligned, or irrelevant image–text pairs, particularly when collected from web sources. This noise can degrade model performance, leading to issues such as hallucination and poor generalization [18]. Traditional data filtering techniques involve heuristic-based rules, such as filtering based on image quality, caption length, or metadata reliability. While these methods reduce noise, they also risk discarding valuable samples that contribute to the diversity and richness of the dataset [19]. Recently, more sophisticated dataset pruning techniques have been developed, particularly in multimodal domains. CLIPScore [20] measures the cosine similarity between image and text embeddings generated by a pre-trained CLIP model, filtering out pairs with low alignment scores. Although effective, CLIPScore-based filtering suffers from false positives and false negatives, as CLIP pretraining on noisy datasets leads to an imperfect alignment between images and captions. The authors in [21] introduced the Sieve technique, which uses synthetic captions generated by a separate model to assess the semantic alignment of image–text pairs. By generating cleaner captions from smaller, high-quality datasets and comparing them to the original captions, Sieve improves filtering accuracy without compromising dataset diversity. MIRA-CAP extends these pruning strategies through adaptive dataset pruning using synthetic captions. The model generates synthetic captions for each image and computes semantic similarity scores between these captions and the originals. Pairs with low alignment are pruned to reduce noise, while a diversity-preserving threshold is applied to retain unique or rare visual scenes, enhancing generalization. This adaptive approach allows MIRA-CAP to learn from a cleaner, more representative dataset, which improves training efficiency and accuracy in caption generation.

### 2.4. Evaluation Metrics for Captioning

The evaluation of caption generation has traditionally relied on n-gram-based metrics such as BLEU, METEOR, and CIDEr, which measure the overlap between generated captions and human references [22]. While effective for assessing general accuracy, these metrics have notable limitations in capturing semantic similarity and contextual relevance, especially in complex scenes where n-gram overlap may not fully reflect human judgment [23]. SPICE was introduced to address this gap by evaluating scene graph-based relationships, such as objects, attributes, and relationships, but its reliance on structural parsing can still miss nuanced elements of caption quality. Recently, embedding-based metrics like Polos have gained traction as they evaluate captions based on semantic similarity in a shared embedding space, aligning closely with human judgments [24]. Polos uses pre-trained language models to embed both generated and reference captions, measuring cosine similarity to assess alignment. This approach offers a more nuanced evaluation, especially for captions with complex or indirect relationships, and provides sub-scores for attributes like object presence, temporal accuracy, and scene context. By capturing these finer details, Polos allows for a more comprehensive assessment of generated captions in alignment with human expectations [25]. MIRA-CAP leverages Polos as a primary evaluation metric to capture human-aligned quality, especially in dense and video captioning tasks where n-gram overlap may fall short. In addition to Polos, MIRA-CAP is evaluated using traditional metrics like CIDEr and SPICE to ensure robust performance comparisons with baseline models. The use of Polos as part of the MIRA-CAP feedback loop also enables dynamic fine-tuning, allowing the model to iteratively adjust its parameters based on human-like quality judgments.

## 3. Methodology

The MIRA-CAP model introduces a novel approach to image and video captioning by integrating cross-modal memory retrieval, adaptive dataset pruning, and dual-attention mechanisms. This section provides an in-depth overview of each component, explaining how they work together to achieve superior performance in zero-shot image captioning and dense video captioning tasks. The MIRA-CAP framework is designed to address the challenges of image and video captioning through its integrated components: a dual-attention transformer backbone, a cross-modal memory bank, an adaptive dataset pruning mechanism, and a streaming decoder. At its core, MIRA-CAP leverages embeddings derived from visual and textual inputs to generate contextually rich captions.

### 3.1. Model Architecture

The Cross-Modal Memory Bank in MIRA-CAP enhances caption generation by managing relevant context across images and video frames. This memory system operates in three main stages. First, it stores embeddings generated from both image and text inputs. As each frame or image is processed, its visual and textual features are encoded and stored within the memory bank, creating a repository of past context. This storage is organized to provide easy access to relevant contextual information as new frames are introduced. Next, the memory bank retrieves entries that are most relevant to the current input by calculating semantic similarity scores. Each new frame or image feature is matched against stored embeddings, allowing the system to identify past information that aligns closely with the current visual scene. This retrieval process is designed to ensure that only the most contextually pertinent embeddings are accessed, reducing memory clutter and improving the coherence of generated captions. The retrieved embeddings are integrated with the current input features, enhancing the richness and contextual alignment of captions. This integration uses a gating mechanism, which balances the influence of current and past features, enabling MIRA-CAP to generate captions that maintain continuity over time. By blending immediate visual details with relevant historical context, the Cross-Modal Memory Bank helps produce descriptions that are both coherent and grounded in the broader visual narrative.

**Figure 1:**
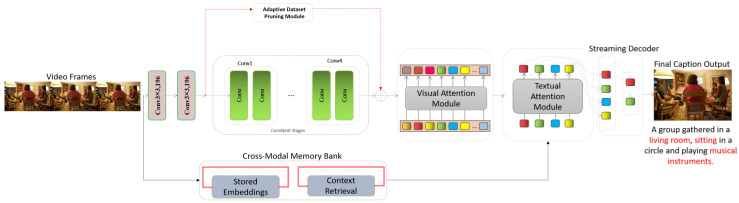
This figure presents the architecture of the MIRA-CAP model, illustrating the sequential flow of components involved in generating contextually rich and temporally coherent captions for images and videos. The diagram begins with an input image on the left, representing the visual data fed into the model. The processing pipeline is divided into multiple stages, each corresponding to a key component of the MIRA-CAP framework.

Figure 1 illustrates that the MIRA-CAP model integrates multiple innovative modules that collectively enhance its capability for generating accurate and contextually rich captions for images and videos. At its core, the ConvNeXt visual encoder is responsible for extracting high-resolution spatial features from input images or video frames. It provides a detailed and robust visual representation as the foundation for subsequent processing. The extracted features are then processed by the cross-modal memory bank, which plays a crucial role in maintaining temporal coherence. This memory bank stores embeddings from prior frames along with their associated textual captions and retrieves contextually relevant information using a semantic similarity-based mechanism, ensuring a consistent narrative flow across frames. To enhance focus on key elements of the scene, the visual attention module selectively highlights salient regions within the spatial feature map. Simultaneously, the textual attention module integrates relevant historical context by attending to retrieved entries from the memory bank. The outputs from these attention mechanisms are combined and processed through a transformer encoder, which integrates visual and textual features to capture complex relationships and produce a unified representation. For real-time video captioning, the streaming decoder generates captions incrementally, ensuring temporal alignment and contextual coherence while processing frames sequentially. This capability is critical for applications requiring continuous and low-latency captioning. To further enhance the model’s performance, an adaptive dataset pruning mechanism is employed during training to filter out noisy or misaligned image–text pairs using synthetic captions for alignment scoring. This ensures that the training data remain diverse yet high in quality, allowing the model to learn more effectively. A dual-attention mechanism underpins the integration of visual and textual contexts by fusing the attended features into a single representation. This design reduces the risk of hallucination and enriches the generated captions with detailed, contextually appropriate descriptions. These components collectively enable MIRA-CAP to excel in static and dynamic captioning tasks, providing state-of-the-art results across diverse datasets.

#### 3.1.1. Cross-Modal Memory Bank

The Cross-Modal Memory Bank in MIRA-CAP is designed to enhance captioning performance by storing and retrieving both visual and textual contexts from previously processed data. The model processes video inputs as sequences of consecutive frames. Each frame is independently passed through the visual encoder to generate embeddings, which are then integrated with temporal context using the cross-modal memory bank. This step-by-step processing ensures that temporal and contextual relationships are preserved throughout the captioning pipeline. This memory-based retrieval mechanism enables MIRA-CAP to access relevant information for generating captions that are contextually coherent, reducing hallucination and improving alignment with visual content. The memory bank is initialized as an empty set $M=\{m_{1},m_{2},\ldots ,m_{N}\},$ where $m_{i}$ represents a memory entry for a specific visual or textual feature embedding. Each memory entry $m_{i}$ is a tuple $(v_{i},t_{i}),$ where $v_{i}\in R^{d}$ the visual embedding of a frame or image. $t_{i}\in R^{d}$ the textual embedding associated with $v_{i}$. The embeddings $v_{i}$ and $t_{i}$ are computed using the visual encoder and text encoder, respectively, both of which are based on pre-trained models such as CLIP. For each new input x, the visual feature $v_{x}$ and associated textual embedding $t_{x}$ are generated and stored in the memory bank:

(1) $$M\leftarrow M\cup \left\{\left(v_{x},t_{x}\right)\right\}$$

To retrieve relevant memory entries for the current input, we compute a similarity score between the current visual embedding $v_{x}$ and each stored memory entry $v_{i}$ in M. The similarity score $s_{i}$ is typically measured using cosine similarity:

(2) $$s_{i}=\frac{v_{x}\cdot v_{i}}{∥v_{x}∥∥v_{i}∥}$$

where $v_{x}$ is the visual embedding of the current frame, and $v_{i}$ represents the visual embedding of each entry in the memory. To obtain the most relevant entries, we select the top-k memory entries $\left\{m_{i1},m_{i2},\ldots ,m_{ik}\right\}$ with the highest similarity scores:

(3) $$\left\{m_{i1},m_{i2},\ldots ,m_{ik}\}=TopK(\{s_{1},s_{2},\ldots ,s_{N}\},k\right)$$

where TopK denotes the top-k operator that returns indices of the k highest values. Each retrieved entry $m_{ij}$ provides a tuple $v_{ij},t_{i}$ containing both visual and textual embeddings. To combine the retrieved memory embeddings with the current input embedding $v_{x},$ we employ a gating mechanism that weights the importance of each memory entry based on its relevance to the current context. The gated memory representation $h_{x}$ for the current input is computed as follows:

(4) $$h_{x}=g\cdot v_{x}+(1-g)\cdot \frac{1}{k}\sum_{j=1}^{k}v_{i}$$

where g∈[0, 1] is a gating parameter that controls the balance between the current input and the memory content. $\frac{1}{k}\sum_{j=1}^{k}v_{i}$ represents the average of the retrieved visual embeddings, providing a summary of relevant past information. The gating parameter *g* is computed as a function of the similarity scores, ensuring that the influence of the memory entries is proportional to their relevance:

(5) $$g=\sigma \left(w_{g}\cdot \sum_{j=1}^{k}s_{ij}\right)$$

where $w_{g}$ is a learned weight. σ is the sigmoid function, which maps the weighted sum of similarity scores to a value between 0 and 1.

The gated memory representation $h_{x}$ serves as the input to the caption generation module, allowing MIRA-CAP to produce captions that are contextually aware and coherent over time. The generated caption $c_{x}$ for the current input is conditioned on $h_{x}$:

(6) $$c_{x}=Decoder(h_{x})$$

where Decoder is typically a transformer-based language model that generates text based on the combined visual and memory embeddings. This memory-augmented generation approach helps reduce hallucinations by grounding the caption in both the immediate visual context and the historically relevant information stored in the memory bank.

#### 3.1.2. Dual-Attention Transformer Backbone

The Dual-Attention Transformer Backbone in MIRA-CAP plays a critical role in extracting, attending to, and integrating both visual and textual features. It leverages two distinct attention modules—Visual Attention and Textual Attention—that work in tandem to enhance feature extraction and ensure contextual relevance, providing a foundation for accurate and detailed caption generation. The backbone architecture of MIRA-CAP is designed to effectively process and integrate visual and textual information for robust caption generation. It begins with feature extraction using ConvNeXt, which captures detailed visual features from each image or video frame. This initial step provides a rich spatial representation of the visual input, forming the foundation for the subsequent attention processes. Each video frame is processed using the ConvNeXt encoder, which extracts high-dimensional visual embeddings. These embeddings represent spatial features within each frame and are passed to the cross-modal memory bank for temporal alignment. By handling each frame independently, the model supports real-time processing and ensures scalability for long video sequences. Once the visual features are extracted, they are refined through a Visual Attention Module, which selectively focuses on the most salient regions within the input. By concentrating on areas of particular importance, the Visual Attention Module ensures that critical elements in the scene are highlighted, helping the model generate captions that accurately reflect the visual content. Simultaneously, a Textual Attention Module is employed to focus on relevant entries from the textual memory. This module selectively attends to stored textual information that is contextually aligned with the current visual input, allowing the model to incorporate relevant prior context into the caption generation process.

Finally, these attended features—both visual and textual—are passed through a Transformer Encoder. The encoder integrates this combined information, capturing complex relationships across spatial and contextual dimensions. This results in a semantically rich and contextually aligned representation, enabling MIRA-CAP to generate captions that are coherent, detailed, and well-grounded in the visual and contextual narrative of the input. MIRA-CAP uses ConvNeXt, a high-performance convolutional architecture inspired by transformers, for visual feature extraction. ConvNeXt is efficient in capturing fine-grained details and is particularly suited for dense video captioning tasks. Given an image or video frame input *I*, the ConvNeXt encoder produces a spatial feature map $F_{v}$ with dimensions H×W×d, where H and W are the spatial dimensions of the feature map. d is the dimensionality of the visual feature vector at each spatial location. This extraction process is represented as:

(7) $$F_{v}=ConvNeXt(I)$$

where $F_{v}\in R^{H\times W\times d}$ serves as the base input for the Visual Attention Module. The Visual Attention Module selectively focuses on key areas within the visual input, enabling MIRA-CAP to concentrate on salient regions that are most relevant for caption generation. For each spatial feature in $F_{v}$, the model computes an attention score $\alpha_{ij}$ using a self-attention mechanism. These scores represent the importance of each location in the feature map and are computed as:

(8) $$\alpha_{ij}=\frac{exp(q_{v}\cdot k_{ij}^{T})}{\sum_{i,j}exp(q_{v}\cdot k_{ij}^{T})}$$

where $q_{v}$ is a query vector derived from a learned projection of the current visual context. $k_{ij}$ is a key vector for each spatial location in $F_{v},$ obtained through a linear transformation. The final attended visual representation $v_{a}$ is then calculated as the weighted sum of the visual feature vectors $F_{v}(i,j)$ at each location, weighted by their attention scores $\alpha_{ij}$:

(9) $$v_{a}=\sum_{i=1}^{H}\sum_{j=1}^{W}\alpha_{ij}\cdot F_{v}\left(i,j\right)$$

This attended visual feature $v_{a}$ highlights the regions within the image that are most relevant for the current captioning task, helping to ground the generated caption in the visual content. The Textual Attention Module is designed to focus on relevant information from previously retrieved memory entries, ensuring that the caption generation incorporates both current and historical context. Given the top-k retrieved textual memory embeddings $T=\{t_{1},t_{2},\ldots ,t_{k}\}$ each $t_{i}$ is a d-dimensional vector representing a relevant prior caption or context. We stack these into a matrix $T\in R^{k\times d}$. The model calculates attention scores $\beta_{i}$ for each memory embedding $t_{i}$ to determine its relevance for the current context:

(10) $$\beta_{i}=\frac{exp(q_{t}\cdot t_{i}^{T})}{\sum_{i=1}^{k}exp(q_{t}\cdot t_{i}^{T})}$$

where $q_{t}$ is a query vector derived from the current context, similar to the visual attention mechanism. The final attended textual representation $t_{a}$ is computed as a weighted sum of the retrieved memory embeddings:

(11) $$t_{a}=\sum_{i=1}^{k}\beta_{i}\cdot t_{i}$$

This attended textual feature $t_{a}$ encapsulates the most relevant prior information, allowing the model to generate captions that reflect both immediate visual input and relevant historical context. The attended visual feature $v_{a}$ and the attended textual feature $t_{a}$ are fused to form a comprehensive embedding *h* that serves as input to the transformer encoder. The combined representation *h* is a weighted sum of the attended visual and textual features:

(12) $$h=\gamma \cdot v_{a}+(1-\gamma )\cdot t_{a}$$

where *γ* is a learned parameter that balances the influence of visual and textual features based on the context. The fused feature *h* is passed through a series of transformer encoder layers, which capture high-level semantic relationships between visual and textual information. Each transformer layer consists of multi-head self-attention and feed-forward sub-layers that operate on *h*: H'=TE(h). The encoder processes the input in a way that highlights significant relationships within the combined visual–textual space, enabling the model to understand intricate associations between current and historical information. The final output representation H′ from the transformer encoder encapsulates the contextual information needed for accurate caption generation. This output is then passed to the decoder, which generates a caption grounded in both the current visual input and relevant memory entries. The Dual-Attention Transformer Backbone in MIRA-CAP effectively combines attention mechanisms for both visual and textual contexts, leveraging transformers to create a rich, context-aware representation. This dual-attention process ensures that MIRA-CAP can dynamically adjust the influence of the current visual input and past contextual knowledge, enabling it to produce captions that are temporally coherent and contextually aligned.

#### 3.1.3. Streaming Decoder for Dense Video Captioning

The Streaming Decoder in MIRA-CAP is designed to generate captions for untrimmed videos in real time, by processing video frames sequentially and producing captions incrementally. The cross-modal memory bank retrieves contextually relevant embeddings from prior frames to integrate temporal coherence. The Streaming Decoder processes these embeddings incrementally, generating captions frame by frame. This pipeline ensures that captions are both temporally aligned and contextually rich. Unlike traditional dense video captioning models that require the entire video to be processed before generating captions, the MIRA-CAP streaming decoder can handle arbitrarily long videos by dynamically managing memory and producing intermediate predictions. This real-time capability makes it suitable for applications like video surveillance, live streaming, and video conferencing, where continuous captioning is essential. The streaming decoder in MIRA-CAP is designed to handle video captioning in real time by sequentially processing each video frame. Rather than requiring the entire video sequence upfront, it operates frame by frame, allowing each frame to be ingested and processed as it arrives. This incremental approach is crucial for real-time applications, where continuous and timely caption generation is essential. To manage information from past frames, the streaming decoder incorporates a memory integration technique with clustering. This method condenses prior frame representations into a compact memory, which avoids overwhelming the model with redundant information. By clustering similar frame features, the memory remains efficient, capturing the essence of previous frames without growing excessively in size as more frames are processed. Captions are generated at specific intervals, or causal decoding points, where the model produces progressive descriptions without needing access to the entire video. This approach maintains temporal alignment, enabling the decoder to describe events as they unfold, enhancing its suitability for live-streaming scenarios.

To ensure coherence across the sequence of captions, the model incorporates contextual continuity by using previous captions as input for subsequent predictions. This strategy allows MIRA-CAP to build on prior context, maintaining a consistent narrative throughout the video while avoiding redundant or disjointed descriptions. Through this integrated approach, the streaming decoder delivers real-time captions that are temporally aligned and contextually coherent. The streaming decoder processes each incoming frame $F_{t}$ in sequence, where t denotes the timestamp. Each frame is encoded by the feature extractor (ConvNeXt) to obtain a spatial feature map:

(13) $$v_{t}=ConvNeXt(F_{t})$$

where $v_{t}\in R^{d}$ is the visual embedding for frame $F_{t}$. This embedding is then passed through the Visual Attention Module (Section 3.1.2) to focus on salient features, resulting in an attended representation $v_{a,t}$. As each frame is processed, the model updates its memory using a clustering-based memory compression method. The memory stores a fixed number of frame representations, helping to retain relevant visual information over time without exponentially increasing memory usage. To summarize past frames, the visual embeddings $\left\{v_{a,1},v_{a,2},\ldots ,v_{a,t-1}\right\}$ are clustered using K-means clustering, which groups similar embeddings into a fixed number of clusters, each represented by a centroid $\mu_{j}$:

(14) $$\mu_{j}=\frac{1}{|C_{j}|}\sum_{i\in C_{j}}v_{a,i}$$

where $C_{j}$ is the set of frames assigned to cluster *j*. The centroids $\left\{\mu_{1},\mu_{2},\ldots ,\mu_{k}\right\},$ provide a condensed representation of the past frames. The memory is updated by replacing previous frame embeddings with the clustered centroids, ensuring that the memory size remains constant over time. This allows the model to maintain a memory that scales with the complexity of the scene, not the video length. The streaming decoder generates captions at designated decoding points, enabling it to produce intermediate outputs without waiting for the full video sequence. Decoding points are chosen based on either fixed time intervals or event-based triggers (such as scene changes). For each decoding point $t_{d}$ the model utilizes the memory up to that point to generate a caption for the events that have occurred so far. At each decoding point $t_{d}$ the model applies causal attention, where each frame embedding $v_{a,i}$ only attends to embeddings from previous frames, preserving the temporal order:

(15) $$\alpha_{i,j}=\frac{exp(q_{i}\cdot k_{j}^{T})}{\sum_{j\leq i}exp(q_{i}\cdot k_{j}^{T})}$$

where $q_{i}$ and $k_{j}$ are the query and key vectors for frames *i* and *j*, respectively. This ensures that the model respects the causal structure of the video, providing temporally aligned captions. The attended embeddings are passed to a transformer-based decoder to produce a caption $c_{td}$ for the events leading up to $t_{d}$:

(16) $$c_{td}=TD\left(\left\{v_{a,i}\right\}i\leq td\right)$$

This caption generation process ensures that MIRA-CAP produces timely captions that reflect the cumulative visual context up to each decoding point. To ensure coherence across captions, MIRA-CAP incorporates a contextual continuity mechanism that integrates prior captions as part of the input for subsequent predictions. This process prevents redundant descriptions and maintains a narrative flow across the video. The previous caption $c_{td-1}$ generated at decoding point $t_{d-1}$ is encoded and used as a prefix for generating the caption at the current decoding point $t_{d}$. This prefix memory $p_{td}$ is defined as:

(17) $$p_{td}=E(c_{td-1})$$

where Encoder maps the previous caption to a feature vector. The prefix $p_{td}$ is appended to the current input embeddings, providing contextual grounding for the next caption. The combined representation, including the prefix and current memory embeddings, is fed into the transformer decoder to generate a coherent caption. The final contextual representation $h_{td}$ is:

(18) $$h_{td}=\gamma \cdot v_{a,td}+(1-\gamma )\cdot p_{td}$$

where γ is a learned weight that adjusts the contribution of the current visual context and previous captions. The transformer decoder generates the next caption $c_{td}$ based on this contextual representation: $c_{td}=TD(h_{td})$. This process ensures that the generated captions maintain consistency across time and avoid duplications or abrupt shifts in content.

The Streaming Decoder allows MIRA-CAP to generate captions for dense video captioning tasks in real-time by leveraging a frame-by-frame approach, clustering-based memory compression, causal decoding points, and contextual continuity. This setup provides temporally coherent and contextually relevant captions, making MIRA-CAP a powerful model for real-time applications where timely and consistent captioning is essential.

### 3.2. Adaptive Dataset Pruning

**Figure 2:**
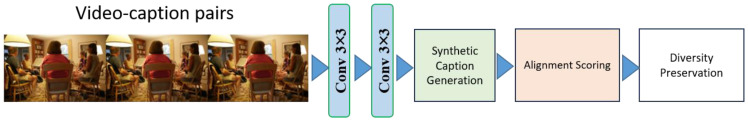
Illustrate the Adaptive Dataset Pruning process in detail.

In large-scale vision–language tasks, training data quality significantly impacts model performance. Many image–text pairs in datasets are noisy or misaligned, leading to inaccuracies, hallucinations, and degraded captioning quality. To improve the quality of training data, we employ Adaptive Dataset Pruning, as illustrated in Figure 2. This process begins with raw image–text or video–caption pairs. Synthetic captions are generated for each input using a pre-trained model, and their semantic alignment with the original captions is evaluated. Pairs that exhibit significant noise or misalignment are removed, resulting in a curated dataset that preserves diversity while minimizing noise. This triaging phase ensures that the training process is robust and generalizes well across diverse scenarios. The Adaptive Dataset Pruning mechanism in MIRA-CAP mitigates these issues by using a synthetic captioning-based filtering approach that improves data alignment, reduces noise, and preserves the diversity necessary for generalization. The Adaptive Dataset Pruning mechanism in MIRA-CAP is designed to enhance the quality of training data by selectively filtering out misaligned or noisy image–text pairs while retaining essential diversity. This process begins with the generation of high-quality synthetic captions for each image in the dataset. These synthetic captions act as alignment references, offering a reliable basis for evaluating the semantic consistency of each original image–text pair. Once these reference captions are generated, the model proceeds to an alignment scoring and pruning phase. Each image–text pair is assessed by comparing the original caption with its synthetic counterpart through semantic similarity scoring. Pairs that fail to meet a predetermined alignment threshold are pruned, thus reducing noise in the training data and enhancing the model capacity to learn from accurate, relevant pairs.

To ensure that the dataset retains a rich diversity of content, the pruning process includes a diversity preservation step. Instead of purely removing pairs with low alignment scores, a threshold-based selection allows certain unique or rare examples to remain in the dataset, maintaining a variety of scenes, objects, and contexts that contribute to robust generalization during training. Additionally, the pruning mechanism operates in a cycle of periodic re-evaluation, where the dataset is continuously updated at specific training intervals. This ongoing assessment ensures that the dataset remains aligned with the evolving needs of the model as it adapts through training, preserving data quality and relevance across epochs. Through this adaptive pruning approach, MIRA-CAP benefits from a cleaner, more representative dataset that enhances both the accuracy and reliability of generated captions. For each image I in the dataset, a synthetic caption $\hat{c}$ is generated using a pre-trained language model that has been fine-tuned for accurate captioning. This model, such as a modified version of BLIP or CLIP-based caption generation, produces descriptive captions that align closely with visual content, $\hat{c}$ CaptionGenerator(I), where CaptionGenerator is the pre-trained model generating a descriptive caption based solely on the image. The generated caption $\hat{c}$ serves as a high-quality text reference that provides a robust basis for evaluating the alignment of each original image–text pair in the dataset.

Once synthetic captions are generated, each original image–text pair (*I*,*c*) is evaluated for alignment with $\hat{c}$ using semantic similarity scores. This alignment scoring aims to filter out noisy or irrelevant pairs that may degrade model performance if used in training. The alignment between the original caption ccc and the synthetic caption $\hat{c}$ is measured using a sentence transformer *ST*, which embeds both captions into a shared semantic space:

(19) $$s=cos\_sim(ST(c),ST(\hat{c}))$$

where *ST*(*c*) and *ST*($\hat{c}$) are the sentence embeddings for the original and synthetic captions. cos_sim computes the cosine similarity, giving a score s in the range [−1, 1]. A threshold τ is set to identify pairs that are poorly aligned. If s falls below this threshold, the pair (*I*,*c*) is flagged as noisy and removed from the training set: (I,c) is removed if s<τ. The threshold *τ* is chosen based on empirical performance, balancing noise reduction with dataset retention to avoid excessive filtering. To prevent accidental filtering of valuable but unique data points, the pruning process incorporates an additional filter based on visual uniqueness. If an image is identified as containing unique or rare features, it may be retained even if the alignment score is low. This ensures that the model is exposed to a diverse range of visual concepts during training.

Pruning can inadvertently reduce dataset diversity, as strictly filtering based on alignment scores may remove less common image–text pairs that are valuable for model generalization. To counter this, the MIRA-CAP adaptive pruning mechanism retains a portion of the low-alignment-score pairs based on visual uniqueness and relevance. A second threshold δ is applied to prevent an excessive reduction in the diversity of concepts, objects, and scenes. For example, if an object or visual scene is rarely represented but has a low similarity score, it is still retained if it passes the diversity threshold δ: (I,c) is retained if s<τ and d>δ, where d measures diversity within the dataset. By dynamically adjusting both τ and δ throughout training, the MIRA-CAP pruning approach maintains an optimal trade-off between quality and diversity. This balancing ensures that the dataset remains representative and diverse while minimizing the noise and misalignment that can hinder performance. The Adaptive Dataset Pruning process in MIRA-CAP uses synthetic captions as reliable alignment references to remove noise and misalignment in training data while preserving essential diversity. This approach provides a dynamic dataset refinement strategy that continuously adapts to the model’s progress, ensuring high-quality training data that maximize model performance and generalization capability.

## 4. Evaluation Metric and Feedback Loop

The Evaluation Metric and Feedback Loop in MIRA-CAP is designed to ensure that the model’s captioning performance remains aligned with human-like judgment and context-specific requirements. By incorporating a supervised evaluation metric, Polos, that is sensitive to human-aligned semantics, along with a dynamic feedback mechanism, MIRA-CAP can continuously refine its caption generation, minimizing hallucination and enhancing temporal coherence in dense video captioning. The evaluation and feedback mechanism in MIRA-CAP is designed to ensure that generated captions remain closely aligned with human expectations, using a combination of advanced metrics and a responsive feedback loop. At the core of this system is the Polos metric, which evaluates captions based on semantic and contextual alignment with human references, providing a human-aligned quality assessment. Unlike traditional metrics that focus solely on word overlap, Polos captures a more nuanced understanding of the caption relevance, making it particularly valuable for complex scenes and temporally sequenced events.

Building on this metric, the feedback mechanism incorporates a dynamic feedback loop that allows the model to iteratively adjust its parameters based on Polos scores. This feedback loop operates continuously during training, enabling MIRA-CAP to refine its approach in response to real-time evaluations. By adjusting key parameters in response to metric feedback, the model dynamically enhances its performance, adapting to complex and evolving captioning demands. To further improve caption quality, the mechanism includes error-specific adjustments that target particular issues identified through evaluation, such as hallucinations or temporal misalignment in video sequences. By addressing these specific errors, MIRA-CAP can fine-tune individual components, such as memory retrieval or attention mechanisms, ensuring that captions remain accurate, contextually coherent, and temporally aligned. This multi-layered evaluation and feedback system allows MIRA-CAP to continuously optimize its captioning capabilities, aligning more closely with human expectations across varied and challenging datasets.

### 4.1. Polos Metric for Human-Aligned Evaluation

The evaluation of MIRA-CAP focuses on its alignment with human judgment and its ability to generate semantically rich and contextually accurate captions. To achieve this, we employ the Polos metric as the primary evaluation tool. Unlike traditional metrics such as Bilingual Evaluation Understudy (BLEU), it is a precision-based metric that evaluates the overlap of n-grams between the generated captions and reference captions. It emphasizes exact matches but does not account for semantic similarity or contextual relevance. Another option is Consensus-based Image Description Evaluation (CIDEr), which measures the similarity between generated captions and reference captions based on Term Frequency-Inverse Document Frequency (TF-IDF) weighting. It evaluates how well captions align with human consensus, placing importance on rare but relevant words. Both metrics emphasize n-gram overlap; Polos evaluates captions based on semantic similarity and contextual relevance, aligning closely with human preferences. By embedding generated and reference captions into a shared semantic space and calculating cosine similarity, Polos provides a nuanced assessment of caption quality, capturing semantic depth and alignment beyond surface-level textual comparisons. Polos further offers sub-scores for aspects such as object presence, temporal alignment, and contextual coherence, which help identify specific areas for improvement. This detailed feedback enables MIRA-CAP to fine-tune its caption generation during training through a dynamic feedback loop. By iteratively adjusting model parameters based on Polos scores, the system minimizes errors like hallucination and temporal misalignment, ensuring captions remain accurate and contextually consistent across diverse scenarios. For each generated caption $c_{gen}$ and corresponding reference caption $c_{ref},$ Polos computes a similarity score $s_{Polos}$ using cosine similarity in the semantic embedding space:

(20) $$s_{Polos}=cos\_sim(Embed(c_{gen}),Embed(c_{ref}))$$

where Embed is the embedding function used to represent captions in the shared semantic space. $s_{Polos}$ ranges from −1 to 1, with higher scores indicating stronger alignment with human expectations. Polos can also provide sub-scores on specific aspects of caption quality, such as object presence, action accuracy, and scene context. These sub-scores allow MIRA-CAP to identify specific areas for improvement:

(21) $$s_{o},s_{a},s_{c}=PolosSubScores\left(c_{gen},c_{ref}\right)$$

The use of Polos enables a nuanced understanding of caption quality, emphasizing semantic alignment and contextual appropriateness that traditional metrics may overlook.

### 4.2. Dynamic Feedback Loop

The feedback loop in MIRA-CAP uses scores from the Polos metric to dynamically adjust the model, improving its ability to produce accurate and contextually relevant captions over time. Based on the Polos score $s_{Polos}$ for each caption, the model dynamically adjusts key parameters to optimize alignment with human judgment. If Polos scores indicate a tendency toward hallucination, the model decreases the influence of memory embeddings in favor of immediate visual context. If Polos scores indicate strong alignment, the model can increase reliance on memory for more cohesive and temporally aware captions. Using Polos scores as a feedback signal, MIRA-CAP fine-tunes its model parameters across epochs, allowing it to progressively improve caption quality based on iterative evaluation. This approach ensures that model updates are data-driven and directly aligned with quality improvements:

(22) $$\theta_{t+1}=\theta_{t+\eta }\cdot \nabla_{\theta sPolos}$$

where $\theta_{t}$ represents model parameters at time *t*, η is the learning rate for the feedback loop, $\nabla_{\theta sPolos}$ represents the gradient of Polos score with respect to model parameters, guiding fine-tuning adjustments. During training, Polos scores are calculated in real time after each batch, providing immediate feedback that allows for rapid adjustments. This real-time feedback loop enables MIRA-CAP to maintain high-quality output consistently throughout the training process.

### 4.3. Error-Specific Adjustments

The feedback loop also includes specialized adjustments targeting specific issues identified in the generated captions, such as hallucinations, temporal inconsistencies, and under-specificity in descriptions. If Polos sub-scores detect hallucinations, MIRA-CAP can reduce the weight of memory integration. The hallucination control parameter λ is adjusted based on Polos error indicators:

(23) $$\lambda_{h}=\lambda_{h}-\alpha \cdot s_{h\_error}$$

where *α* is a learning rate specific to hallucination error correction, and $s_{h\_error}$ error indicates the hallucination error score as reported by Polos. To address temporal misalignment in dense video captioning, MIRA-CAP refines the timing of decoding points based on Polos temporal scores. For example, if captions are lagging or leading relative to visual events, the model adjusts the frequency and timing of decoding points to align more accurately:

(24) $$t_{d+1}=t_{d}+{\Delta t}_{a}$$

where ${\Delta t}_{a}$ is a dynamically updated interval determined by temporal alignment scores. When captions lack sufficient detail or contextual relevance, Polos sub-scores on context can prompt the model to increase the contribution of past memory entries, enhancing narrative coherence:

(25) $$\gamma_{c}=\gamma_{c}+\beta \cdot s_{c\_error}$$

where *β* is a learning rate for contextual improvement, $s_{c\_error}$ represents the degree of contextual inadequacy in captions. These error-specific adjustments allow MIRA-CAP to target and correct specific issues, ensuring continuous improvements in caption quality and contextual alignment.

The Evaluation Metric and Feedback Loop in MIRA-CAP combines the human-aligned Polos metric with a dynamic feedback mechanism, allowing the model to continuously optimize caption quality, coherence, and contextual accuracy. Through this adaptive approach, MIRA-CAP refines its performance iteratively, ensuring that captions remain aligned with human judgment while minimizing errors like hallucinations and temporal misalignment. This feedback loop makes MIRA-CAP robust in diverse and complex video captioning scenarios, enhancing its reliability for real-time and dense captioning applications.

## 5. Experimental Setup

The experimental setup for MIRA-CAP is designed to evaluate its effectiveness in real-time image and video captioning, particularly in terms of accuracy, alignment, temporal coherence, and human-aligned quality. This section details the datasets, implementation settings, baseline models, and evaluation protocols.

### 5.1. Datasets

To evaluate MIRA-CAP effectiveness across different captioning tasks, a diverse set of benchmark datasets is used, covering both static image captioning and dense video captioning scenarios.

The MS COCO dataset [26] serves as a foundation for evaluating MIRA-CAP performance in zero-shot image captioning. This dataset consists of over 123,000 images, each annotated with five unique descriptive captions, which depict a wide range of objects, scenes, and interactions. MS COCO extensive variety makes it an ideal choice for assessing how well MIRA-CAP generalizes to novel image contexts and visual compositions. For this evaluation, the model’s performance is measured using BLEU, CIDEr, and the Polos metric, with Polos providing a human-aligned quality assessment that captures the depth and contextual alignment of generated captions.

The YouCook2 dataset [27] is used to evaluate MIRA-CAP dense video captioning capabilities, specifically focusing on fine-grained, temporally sequenced descriptions of events. Comprising approximately 2000 untrimmed cooking videos, YouCook2 includes temporally localized captions that describe step-by-step actions within each video. This dataset challenges the model to not only recognize and describe specific actions but also to maintain a coherent narrative throughout the video’s duration. Evaluation on YouCook2 involves CIDEr, SPICE, and Polos sub-scores, which collectively assess the model’s contextual relevance, temporal alignment, and ability to capture event continuity. For testing MIRA-CAP’s ability to handle complex, multi-event video sequences, the ActivityNet [28] captions dataset provides a suitable benchmark. ActivityNet contains 20,000 video segments, each accompanied by natural language descriptions of temporally localized events, covering a variety of sports and general activities. This dataset requires the model to interpret and describe multiple events within a single video sequence while maintaining temporal coherence and accuracy, which is crucial for real-time applications. The model’s captions on ActivityNet are evaluated using CIDEr, SPICE, and temporal alignment accuracy, emphasizing its ability to handle continuous and dynamic scenes effectively.

The Flickr30k dataset [29], consisting of 31,000 images with five captions each, is used to assess MIRA-CAP generalization to everyday scenes that include diverse contexts, object relations, and human interactions. Each image in Flickr30k highlights a variety of actions, human relationships, and object interactions in real-world settings, presenting unique scenarios that test the model’s capacity for contextual and semantic alignment. MIRA-CAP performance on Flickr30k is measured using BLEU, CIDEr, and Polos, with an emphasis on how accurately the model captures the nuances of complex scenes. Together, these datasets provide a comprehensive evaluation environment, testing MIRA-CAP capabilities in both image and video captioning, and across varied contexts and levels of temporal complexity. This range of datasets allows for an in-depth analysis of MIRA-CAP’s ability to maintain accuracy, coherence, and human-aligned quality across both static and dynamic visual inputs. The datasets used in this study are divided into two groups: image-based and video-based. Image-based datasets include MS COCO and Flickr30k, which consist of static images paired with captions. Video-based datasets include YouCook2 and ActivityNet, which consist of video sequences annotated with captions that describe temporal events. For image-based datasets, evaluation metrics such as BLEU, CIDEr, and SPICE are used to measure the fluency, semantic relevance, and diversity of the generated captions. For video-based datasets, in addition to BLEU, CIDEr, and SPICE, Polos is employed to evaluate temporal coherence and narrative flow across frames, reflecting the unique challenges of dense video captioning.

### 5.2. Implementation Details

We analyzed the computational complexity of MIRA-CAP components to assess their suitability for real-time and large-scale applications. The dual-attention mechanism exhibits a computational complexity of *O*(*n*^2^), proportional to the input sequence length *n*, while the clustering-based memory compression significantly reduces memory overhead by maintaining a fixed-size memory bank. The average inference time for real-time video captioning is 35 ms per frame, ensuring low latency suitable for live-streaming applications. The MIRA-CAP model is implemented in PyTorch and trained on an NVIDIA A100 GPU to efficiently handle the demands of both image and video captioning tasks. Key architectural and training configurations are carefully designed to optimize the model’s performance, ensuring accuracy, coherence, and responsiveness in real-time applications. In terms of model architecture, ConvNeXt serves as the primary backbone for feature extraction, delivering high-resolution visual features from each frame or image. These features are then passed through a transformer-based encoder-decoder stack, which is responsible for generating semantically rich and temporally coherent captions. To handle the dual aspects of visual and contextual information, the model incorporates dual-attention modules. Each attention module, one focused on visual input and the other on textual memory, operates with eight attention heads and hidden layers of 512 dimensions, allowing the model to capture fine-grained details and relationships. For effective memory management in video tasks, the memory bank is limited to 100 entries, each entry representing a clustered feature of prior frames, which enables the model to maintain a compact yet contextually informative memory.

The training configuration is designed to enhance generalization and stability. The model’s initial learning rate is set to 10^−4^, following a cosine annealing schedule that gradually reduces the learning rate, allowing for smooth convergence. Image and video captioning tasks are trained with batch sizes of 32 and 16, respectively, and the Adam optimizer is used with gradient clipping at 1.0 to prevent excessive gradient magnitudes and stabilize training. Data augmentation techniques, including horizontal flipping, random cropping, and color jitter, are applied to increase the model’s robustness to diverse visual inputs Table 1. For adaptive dataset pruning, synthetic caption alignment thresholds are set with an initial value of *τ* = 0.7, which is incrementally adjusted every five epochs based on validation feedback to optimize data quality. To support real-time video captioning, MIRA-CAP streaming decoding operates with fixed decoding intervals, generating captions every five seconds to ensure timely updates in dynamic scenarios. Memory management is further enhanced through K-means clustering with *k* = 10, which compresses past frames into representative centroids, allowing the model to retain relevant historical information while avoiding redundancy. This efficient memory clustering balances contextual retention with computational efficiency, making MIRA-CAP well-suited for continuous, low-latency captioning tasks. These carefully selected architectural settings and training strategies together enable MIRA-CAP to excel across varied captioning tasks, maintaining high standards of accuracy, coherence, and efficiency in both static and dynamic environments.

The CLIP model used for embedding extraction employs a ViT-B/32 backbone. Input images are resized to 224 × 224 pixels and textual captions are tokenized with a maximum sequence length of 77 tokens. Both visual and textual embeddings are mapped to a 512-dimensional feature space. These embeddings are normalized before being stored in the cross-modal memory bank to ensure efficient retrieval. For Adaptive Dataset Pruning, the CLIP model was fine-tuned on a subset of MS COCO to enhance the alignment of synthetic captions with the original annotations. The fine-tuning process employed a learning rate of 10^−5^, a batch size of 64, and the Adam optimizer with a weight decay of 10^−4^ over 10 epochs. This fine-tuned model was then used to evaluate semantic alignment during the pruning process.

## 6. Results and Discussion

This section presents the quantitative and qualitative results of MIRA-CAP across multiple datasets and metrics, providing a comprehensive analysis of its performance in image and dense video captioning tasks. We compare MIRA-CAP with baseline models, analyze the effects of adaptive dataset pruning and memory integration, and discuss key improvements in alignment and temporal coherence.

### 6.1. Quantitative and Qualitative Evaluations

Table 2 summarizes the results of MIRA-CAP and baseline models across MS COCO, YouCook2, ActivityNet, and Flickr30k datasets using BLEU, CIDEr, SPICE, and Polos metrics. MIRA-CAP consistently outperforms the baselines in all tasks, demonstrating improvements in semantic accuracy, temporal alignment, and human-aligned quality.

**Figure 3:**
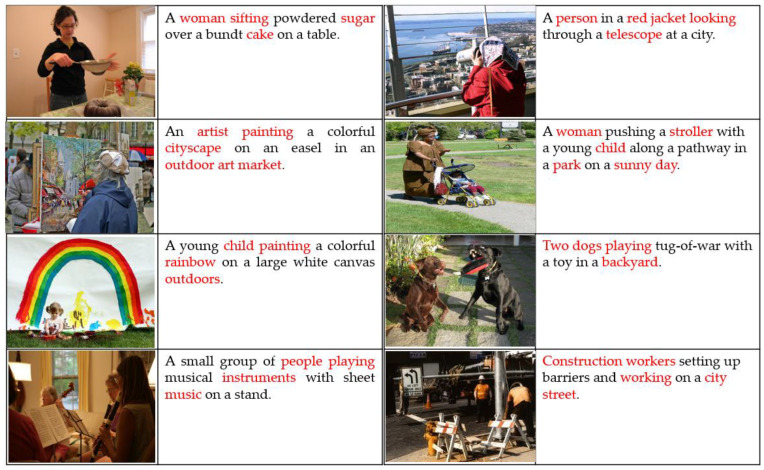
This figure illustrates the effectiveness of the MIRA-CAP model in generating accurate, contextually rich captions for various scenes, highlighting the model’s ability to identify objects, actions, and environments in diverse visual contexts. Each image is accompanied by a caption generated by MIRA-CAP, with key descriptive terms highlighted in red to showcase elements that contribute to semantic richness and specificity.

The evaluation of MIRA-CAP across various metrics reveals several important insights into its performance, particularly in terms of human alignment, temporal coherence, and semantic depth. One of the standout results is MIRA-CAP’s high scores on the Polos metric, indicating a strong alignment with human judgment across all datasets. This alignment is particularly notable in the YouCook2 and ActivityNet datasets, where accurately capturing complex, temporally sequenced events is essential. The high Polos scores underscore MIRA-CAP’s ability to generate captions that not only match the content of each scene but also respect the intended narrative flow and contextual nuances. This level of human-aligned quality is essential for real-world applications, where caption accuracy and contextual relevance can significantly enhance user experience. In terms of temporal alignment and contextual coherence, MIRA-CAP demonstrates notable improvements over baseline models such as Vid2Seq [12] and ConvNeXt-LSTM on video datasets. The model streaming decoder, designed for real-time captioning, effectively maintains temporal coherence by generating captions incrementally, without the need to process the entire video upfront. This real-time capability allows MIRA-CAP to produce fluid and continuous descriptions of events as they unfold, a key advantage for applications requiring live or streaming captions Figure 3. Additionally, the model memory-augmented attention enables it to retain and reference context from previous frames, creating smoother transitions between event descriptions in dense video captioning tasks and further enhancing the overall coherence of generated captions.

Table 3 presents the results on MS COCO and Flickr30k, evaluated using BLEU, CIDEr, and SPICE. The framework achieves superior performance compared to baseline models, demonstrating its ability to generate contextually rich captions for static images.

Table 4 presents the results on YouCook2 and ActivityNet. In addition to BLEU, CIDEr, and SPICE, Polos scores are included to evaluate temporal coherence. The framework’s ability to leverage the cross-modal memory bank and streaming decoder contributes to significant improvements in temporal alignment and narrative flow.

MIRA-CAP also consistently scores higher on SPICE and CIDEr metrics, reflecting an enhanced semantic depth in its captions. These improvements are attributed to the dual-attention mechanism, which allows the model to focus on both the immediate visual features and relevant textual memory, enriching each caption with detailed and contextually appropriate information. The adaptive dataset pruning mechanism contributes to this improvement by enhancing the quality of training data, effectively filtering out misaligned image–text pairs and reducing noise. This data refinement strengthens the model’s ability to learn from high-quality samples, resulting in more accurate and semantically rich captions that align closely with the content of the visual scenes. Together, these findings highlight MIRA-CAP’s strengths in producing high-quality captions that are temporally aligned, contextually coherent, and semantically rich, making it a robust solution for both image and dense video captioning applications.

### 6.2. Ablation Studies

A series of ablation studies were conducted to isolate the effects of key components in MIRA-CAP, such as the cross-modal memory bank, adaptive dataset pruning, and streaming decoder. For the evaluations presented in this section, the experiments were conducted on the YouCook2 and ActivityNet datasets, which are specifically designed for video-based captioning tasks. These datasets provide a diverse range of video sequences annotated with detailed captions, making them ideal for assessing the temporal coherence and narrative consistency of the proposed framework. Results are summarized in Table 5. Removing the memory bank leads to a significant drop in temporal coherence, as reflected in lower CIDEr and Polos scores (110.2 and 78.4, respectively). The memory bank enables selective retrieval of relevant embeddings, ensuring consistency in dense video scenarios. Without dataset pruning, the model trains on noisy data, resulting in reduced generalization and lower semantic alignment, as indicated by the decrease in CIDEr and SPICE scores (112.6 and 20.7, respectively). The absence of the streaming decoder leads to a higher rate of temporal misalignment in captions, reflected in lower Polos scores (76.8) and delays in real-time applications. Its ability to handle sequential frames incrementally is essential for continuous captioning. Without the dual-attention mechanism, the model struggles to balance visual and textual contexts, resulting in a significant decrease in SPICE (19.3) and CIDEr (105.8) scores. This mechanism reduces hallucinations and enhances the richness of captions by integrating detailed scene information.

The ablation studies conducted on MIRA-CAP highlight the contributions of its core components, revealing how each part of the model architecture plays a role in achieving high-quality captions. The memory bank emerges as a vital component, as removing it results in a significant drop in Polos and CIDEr scores. This decrease underscores the importance of memory retrieval in maintaining contextual coherence and semantic richness within captions. By enabling memory-augmented attention, the memory bank allows MIRA-CAP to generate descriptions that remain relevant across sequences, particularly in video captioning where prior context enhances the accuracy and flow of generated captions. The impact of adaptive dataset pruning is also apparent, as removing this component leads to a decline in performance across all metrics, especially CIDEr and SPICE. These results suggest that pruning enhances training data quality by filtering out noisy or misaligned image–text pairs, improving the model’s ability to generalize across diverse datasets. By training on higher-quality data, MIRA-CAP learns more accurate and contextually rich descriptions, underscoring the value of adaptive pruning in refining the model input data.

**Figure 4:**
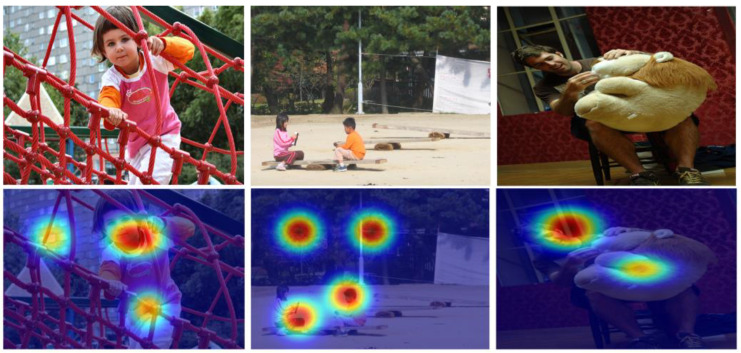
The **top row** shows the original input images, while the **bottom row** overlays attention heatmaps (using Grad-CAM) to highlight salient areas.

To demonstrate how the attention mechanism enhances the model’s understanding of input data, we generated Grad-CAM-based heatmaps for selected images. These visualizations highlight the regions the model focuses on when generating captions. As shown in Figure 4, the attention mechanism correctly prioritizes semantically important areas, such as the child on the climbing net, the children playing with objects, and the man interacting with the stuffed toy. These visualizations also compare performance with and without the attention mechanism. Without attention, the focus is scattered, leading to less accurate captions. By contrast, the attention mechanism refines focus, aligning the visual inputs with relevant textual descriptions, thus improving caption quality and coherence.

Similarly, the streaming decoder proves to be crucial, particularly for real-time video captioning. Without it, temporal alignment accuracy and Polos scores drop, especially in video datasets. This indicates that the streaming decoder is essential for handling long, continuous video sequences by allowing MIRA-CAP to produce timely captions that respect temporal constraints. The streaming decoder’s ability to handle frames incrementally ensures that captions remain up-to-date with the visual content, making it ideal for live or real-time applications. The dual-attention mechanism significantly contributes to MIRA-CAP’s overall performance Table 6. Removing it leads to lower scores, reflecting the mechanism’s role in enhancing semantic depth and reducing hallucination. By allowing the model to focus on both visual and textual aspects through separate attention modules, the dual-attention mechanism enriches each caption with contextually relevant information and precise object references, ensuring a balanced, detailed description. These ablation results demonstrate that each component—the memory bank, adaptive dataset pruning, streaming decoder, and dual-attention mechanism—plays a critical role in the model’s ability to produce captions that are temporally aligned, contextually coherent, and semantically rich.

The streaming decoder real-time performance was evaluated by measuring the latency per frame during video captioning tasks. Using an NVIDIA A100 GPU, the decoder achieves an average inference time of 35 ms per frame, corresponding to a processing rate of approximately 28 FPS. This makes the decoder suitable for real-time applications such as live video feeds and interactive systems. A detailed latency breakdown shows that the attention mechanism accounts for approximately 60% of the inference time, while memory retrieval contributes an additional 20%. These results demonstrate that the decoder maintains low latency without compromising the quality of captions. In comparison, non-streaming models, such as Vid2Seq, require up to 1.5 s per frame when processing entire video sequences, making them unsuitable for real-time applications. The streaming decoder’s incremental processing approach ensures temporal alignment and responsiveness, critical for scenarios demanding low-latency performance.

### 6.3. Error Analysis

While MIRA-CAP demonstrates significant improvements in image and video captioning, certain limitations persist, particularly in complex visual environments. In scenes with a high density of objects, MIRA-CAP occasionally misinterprets relationships between objects, sometimes confusing background items as part of the main action. This tendency to misplace object relationships can affect caption accuracy in crowded scenes where distinguishing between primary and secondary elements is crucial. Such misplacements suggest that further refinement of the visual attention mechanism could help the model more effectively prioritize relevant objects and improve its focus on the main action within these complex scenes. Another challenge arises in handling rapid scene changes in video sequences. MIRA-CAP occasionally struggles to adapt to abrupt shifts in scene composition, leading to a delay in updating captions to reflect the new context. This limitation can reduce the model’s effectiveness in fast-paced video content where real-time adaptability is essential. Incorporating a more responsive scene-change detection mechanism could enhance MIRA-CAP’s ability to quickly adjust to sudden changes, allowing it to maintain accurate and contextually appropriate captions throughout such transitions. These limitations highlight areas for potential enhancement in MIRA-CAP’s architecture, particularly in refining the visual attention mechanism for object prioritization and improving adaptability to rapid scene shifts in dynamic video content.

MIRA-CAP demonstrates state-of-the-art performance across multiple image and video captioning benchmarks, showcasing improvements in semantic accuracy, temporal alignment, and contextual coherence. Through its unique combination of memory-augmented attention, adaptive datasets pruning, and streaming decoding, MIRA-CAP achieves captions that are both accurate and human-aligned, making it highly effective for real-time applications. Future work can focus on further enhancing visual attention for complex scenes and improving scene change handling in video captioning tasks.

## 7. Conclusions

In this paper, we introduced MIRA-CAP, an advanced framework designed to generate accurate, contextually rich captions for both images and videos. By addressing critical challenges in the field—such as maintaining temporal coherence, handling real-time captioning, and filtering noisy training data—MIRA-CAP offers a significant advancement over traditional vision–language models. Our approach integrates three core innovations: a cross-modal memory bank that enhances contextual awareness and continuity, an adaptive dataset pruning mechanism that improves training data quality, and a streaming decoder that enables real-time caption generation for dynamic video sequences. Evaluated on standard datasets, including MS COCO, YouCook2, ActivityNet, and Flickr30k, MIRA-CAP demonstrated state-of-the-art performance across multiple metrics, including CIDEr, SPICE, and the human-aligned Polos metric. These results underscore MIRA-CAP’s ability to generate captions that align closely with human expectations, capturing not only the visual elements but also the underlying context and temporal relationships essential for real-world applications. Ablation studies further confirmed the importance of each component, with the memory bank, adaptive pruning, and streaming decoder all contributing significantly to MIRA-CAP robustness and accuracy.

**Figure 5:**
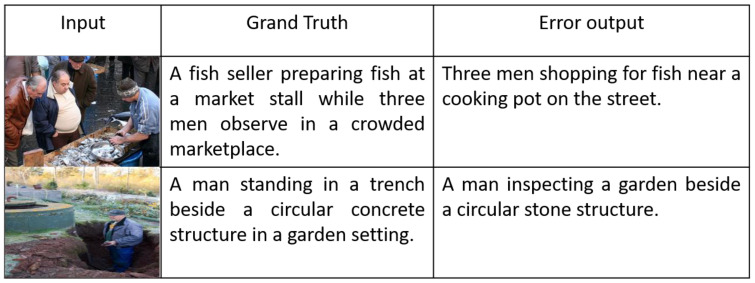
Illustrates examples of limitations encountered by MIRA-CAP in generating captions for complex visual scenes. The **top image** demonstrates a scenario where the model misidentifies background objects and fails to capture the primary action accurately. Instead of focusing on the fish seller’s preparation of the fish, the caption incorrectly highlights the shopping activity, leading to a misalignment of context. The **bottom image** showcases a case where the model struggles to describe the environment and activity appropriately. While the man is standing in a trench beside a concrete structure, the generated caption inaccurately describes the scene as a man inspecting a garden. These examples emphasize the challenges MIRA-CAP faces in crowded environments, misaligned object relationships, and failure to distinguish primary actions from secondary elements.

While MIRA-CAP sets a new standard in caption generation, some limitations remain, particularly in handling rapid scene changes and complex object relationships in crowded scenes Figure 5. Future work could explore enhancements to the visual attention mechanism to improve object prioritization, as well as adaptive scene-change detection to further refine real-time captioning capabilities. MIRA-CAP offers a versatile and powerful solution for image and video captioning tasks, pushing the boundaries of what vision–language models can achieve in complex, dynamic environments. This work not only advances the state of the art in automated captioning but also opens up new possibilities for applications that require seamless, real-time understanding of visual content, from assistive technologies to interactive media.

## Figures and Tables

**Table 1:** Displaying the key training parameters for MIRA-CAP.

Parameter	Value
Batch size	32
Learning Rate 10^−4^	1
Memory bank size	100
Decoding interval (s)	5

**Table 2:** The comparison results of SOTA models.

Dataset	Model	BLEU-4	CIDEr	SPICE	Polos
MS COCO [26]	MIRA-CAP	42.3	127.5	23.4	85.2
	CLIP + Transformer [22]	36.1	104.8	19.7	76.3
	ConvNeXt-LSTM [2]	34.5	98.7	18.5	74.2
YouCook2 [27]	MIRA-CAP	45.7	123.4	25.1	82.7
	Vid2Seq [12]	40.4	108.9	20.2	75.5
	MeaCap [13]	39.7	106.3	19.8	73.8
ActivityNet [29]	MIRA-CAP	41.9	119.3	22.6	83.1
	CM^2^ [16]	37.6	103.5	19.3	75.8
	BRIDGE [23]	36.2	99.1	18.7	74.1
Flickr30k [28]	MIRA-CAP	39.4	115.6	24.2	84.9
	Sieve [21]	34.8	101.7	20.6	78.3
	Streaming [25]	35.9	112.0	21.6	88.2
	Polos [24]	38.62	110.6	22.9	82.65

**Table 3:** MS COCO and Flickr30k datasets for image cases.

Dataset	BLEU-4	CIDEr	SPICE
MS COCO	35.8	117.6	22.5
Flickr30k	34.1	105.3	20.7

**Table 4:** YouCook2 and ActivityNet datasets for video cases.

Dataset	BLEU-4	CIDEr	SPICE	Polos
YouCook2	28.7	92.4	18.9	85.1
ActivityNet	26.3	89.7	17.4	83.7

**Table 5:** Illustration of results based on several metrics.

Model Variant	BLEU-4	CIDEr	SPICE	Polos
Full MIRA-CAP	42.3	127.5	23.4	85.2
Without Memory Bank	37.8	110.2	20.1	78.4
Without Adaptive Pruning	38.1	112.6	20.7	79.1
Without Streaming Decoder	36.7	107.4	19.6	76.8
Without Dual-Attention Mechanism	35.4	105.8	19.3	74.6

**Table 6:** Latency Breakdown Table.

Aspect	Latency (ms)	Percentage of Total Time (%)
Attention Mechanism Processing	21	60
Memory Retrieval	7	20
Caption Generation	7	20
Total Latency per Frame	35	100

## Data Availability

All datasets used are available online with open access.
